# What do medical students think about their quality of life? A qualitative study

**DOI:** 10.1186/1472-6920-12-106

**Published:** 2012-11-05

**Authors:** Patricia Tempski, Patricia L Bellodi, Helena BMS Paro, Sylvia C Enns, Milton A Martins, Lilia B Schraiber

**Affiliations:** 1 Universidade de São Paulo, Av. Dr. Arnaldo, 455 – Cerqueira César, São Paulo, SP, 01246-903, Brazil; 2 Universidade Federal de Uberlândia, Av. Pará, 1720 - Umuarama, Uberlândia, MG, 38408-144, Brazil

**Keywords:** Quality of life, Student, Medical, Medical education

## Abstract

**Background:**

Medical education can affect medical students’ physical and mental health as well as their quality of life. The aim of this study was to assess medical students’ perceptions of their quality of life and its relationship with medical education.

**Methods:**

First- to sixth-year students from six Brazilian medical schools were interviewed using focus groups to explore what medical student’s lives are like, factors related to increases and decreases of their quality of life during medical school, and how they deal with the difficulties in their training.

**Results:**

Students reported a variety of difficulties and crises during medical school. Factors that were reported to decrease their quality of life included competition, unprepared teachers, excessive activities, and medical school schedules that demanded exclusive dedication. Contact with pain, death and suffering and harsh social realities influence their quality of life, as well as frustrations with the program and insecurity regarding their professional future. The scarcity of time for studying, leisure activities, relationships, and rest was considered the main factor of influence. Among factors that increase quality of life are good teachers, classes with good didactic approaches, active learning methodologies, contact with patients, and efficient time management. Students also reported that meaningful relationships with family members, friends, or teachers increase their quality of life.

**Conclusion:**

Quality of teachers, curricula, healthy lifestyles related to eating habits, sleep, and physical activity modify medical students’ quality of life. Lack of time due to medical school obligations was a major impact factor. Students affirm their quality of life is influenced by their medical school experiences, but they also reframe their difficulties, herein represented by their poor quality of life, understood as necessary and inherent to the process of becoming doctors.

## Background

Medical education has been shown to be hazardous to students’ health [1] and create an environment of psychological toxicity [2-4]. Medical students present higher levels of stress when compared with other young people of the same age in other programs [5]. They also have higher scores of daytime somnolence [6-9]. Getting into medical school has an impact on a student’s health and quality of life because it requires adaptation and lifestyle changes [9]. Studies with medical students have found that during the first year of medical school students had a deficit in hours of sleep, physical activity, and social interactions [10,11]. Knowing the factors that influence students’ quality of life during medical training facilitates healthcare promotion and psycho-pedagogical support services. It is also important for curriculum design.

Quality of life has been defined by the World Health Organization (WHO) in its multicenter studies as, “the individual’s perception of his position in life, within the context of culture and system of values wherein the individual lives and in relation to his objectives, expectations, standards and concerns” [12]. Although WHO defined what quality of life is, they didn’t establish the minimum level of quality of life, considering age, gender, occupation, and culture [13]. Therefore, the perception of quality of life is individual, subjective, and temporary, which makes its measurement and comparisons difficult [12-14].

Considering the difficulty in assessing quality of life, we believe that to understand medical students’ quality of life it is important to know their learning environment and lives in medical school by giving voice to their concerns.

The objectives of this study were to assess medical students’ perception of their quality of life and to explore its modifying factors related to the academic environment and individual skills.

## Methods

### Participants

This was a qualitative study with focus groups consisting of a convenience sample of medical students from the first to sixth year of training at six medical schools from different regions in Brazil. Medical schools were chosen to represent all medical education designs in Brazil. Schools comprised a public and a private school, a school with a problem-based learning (PBL) curriculum, a school with a traditional curriculum, a school in a capital city, and a school in the countryside. As we had data saturation with the participation of seven schools, six schools were included in this study. A total of 56 students were interviewed in Portuguese after signing written informed consent.

### Instruments

Focus groups were based on a semi-structured interview, with open-ended questions about quality of life, its modifying factors, and coping strategies. Students in focus groups were asked the following questions: “*What is the concept of quality of life in your view*?”, “*What is a medical student’s quality of life like*?”, “*What are the factors that increase quality of life in medical school*?”, “*What are the factors that decrease quality of life in medical school*?”, “*What are the coping strategies you use to improve your quality of life*?” Two researchers performed group coordination, one acting as the coordinator and the other as an assistant. At the beginning of each discussion, the coordinator welcomed participants, provided information about the objectives and methodology of focus groups, and warranted confidentiality. The coordinator also promoted participation and concluded the meeting by giving feedback on the discussed themes and by guaranteeing access to study results.

Following recommended methodology for focus groups [15-18], sessions lasted 90–120 minutes and were carried out inside the medical schools, in a private, silent and comfortable environment, allowing visual contact among all participants.

### Procedures

Three medical students from each year of training (1^st^ to 6^th^) were randomly recruited by telephone contact to compose focus groups (n=108). Of these, 56 students were able to attend the sessions, which ranged in size from 6–15 participants each. Groups were heterogeneous regarding gender and year of medical training. All discussions were audio taped.

### Data analysis

Audio recordings of the six focus groups were transcribed for analysis and categorized according to traditional methods of content analysis [16-18]. Analysis started with a free reading of the transcribed text by two independent researchers. This initial reading aimed at impregnating researchers with the study topic without any intention of categorization. During a second reading of the transcripts the two researchers performed categorization of emerging themes and derived issues independently. Each researcher’s results were paired by similarity of meaning and reviewed by the entire research team. Excerpts from group interviews were chosen to illustrate each theme and were then translated into English.

## Results

Several themes emerged from the analysis, as follows:

1. What a medical student’s life is like

Students see the medical profession as one that brings power and status. They experienced, as early as the beginning of the medical program, feelings of omnipotence reflecting the role of physicians in society. However, at the same time, they faced situations of pain, suffering, death and social realities different from their own. They experienced an ambivalence of feeling proud coupled with the fears, demands, pressure, and insecurities of their profession. They considered that a medical program demands exclusive dedication and indeed they dedicated a large part of their time to it, to the detriment of other areas of their lives. When they compared themselves to other young people from other programs, they felt that more is demanded from them. They also felt more stressed, more responsible, and less capable of managing their personal and financial lives. Considering these ambivalent experiences, students must learn to leave this “mesh” and differentiate good from bad experiences in academic life. Thus, in the midst of this mix of sacrifice and renouncement, they also recognized rewards and satisfaction (Table 1). Participants reported:

“There is a power in the profession, which has an effect on patients’ lives. This power is theoretical and practical; thus, some people feel as if they were demigods.”

“It is like having a baby, you know? The baby steals your personal life, your time, your girlfriend, your hours of sleep and occupies space in your house. However, you are completely crazy for him or her. This is the same feeling in medical school, it is great.”

“‘You look like hell’ (my friend said)…and I answered, ‘Thanks! I did not sleep last night and was in college the whole day.’ Well, she is getting married and I don’t even have time to get a boyfriend.”

“I think that being a medical student is really quite different from being a student in any other program. You are, from the very beginning, learning to live with situations where patients are very sick, almost dying, or other situations that are psychologically very stressful. So, I think that it makes you mature quicker.”

Participants also frequently reported competitiveness among peers due to concerns related to continuing education in highly-requested residency programs (usually those with specialization in areas of potentially better income).

“The competition is big for monitoring, clerkship and residency.”

2. Concepts of quality of life for medical students

Students understand quality of life as a multidimensional construct comprising happiness and life satisfaction, healthy habits, social and affective relationships, and freedom. Time management emerged as an important defining factor for participants’ quality of life.

“Quality of life is being able to manage time well.”

“Quality of life is being able to accomplish my goal in life.”

Table 2 shows other examples of categories and issues for this theme.

3. Factors that increase quality of life in the medical program

Among factors that increase quality of life in medical education are those associated with pedagogical aspects of the program: good teachers, didactically taught classes, and well-designed assessments. Participants reported that participating in social development projects and scientific research also increases quality of life, because they improve their self-esteem and usefulness as they carry out their social role. When asked about teaching methodology, students in a PBL curriculum reported being more satisfied with their quality of life than those following a traditional approach. Perhaps this better perception about quality of life among PBL students is related to the spare time they have to study. They have also more freedom and autonomy to manage their time.

Participants reported that contact with patients makes them feel useful and contextualizes the content they learn. They stated that clerkship (5^th^ and 6^th^ years of the medical program) is the most stimulating part of the program and also requires the most dedication. This period is characterized by a great deal of learning and responsibility. Because this phase deals with students’ professional future, insecurities regarding the profession are the highest.

“Contact with patients makes you feel useful and contextualizes the study.”

“The internship is the most stimulating period. You have a team, get stimulated, want to study and see everything. If the whole program were like that, we would be much better doctors.”

Participants also reported that quality of life improves when one has significant relationships with family, friends, or tutors-mentors. They also said that managing their time well and having personal and academic activities improves quality of life.

“Having breakfast with my mother increases my quality of life.”

“What improves quality of life is the attitude you have regarding your life and your program. The question is to make the best use of your time, separating time for the things you like. You should think: I am not only a medical student; I am a citizen with a girlfriend, family and friends. If you recognize that, you will organize your time better, you will try to do things you like at the right moment and try not to demand so much from yourself. And you live better, in the program or in spite of it.”

Table 3 shows other examples of categories and issues for this theme.

4. Factors that decrease quality of life in the medical program

The factors that decrease students’ quality of life are associated with the learning environment. Bad teachers and coordinators, and classes without a didactic approach worsen quality of life. Being under pressure or moral abuse by classmates, teachers, or patients are other factors that decrease quality of life.

In almost all participants’ reports, the main factor of a reduced quality of life was related to lack of time. Some participants reported a lack of care with their own health according to frequently harmful habits adopted by residents and teachers.

“I learned with a resident to put lots of sugar in coffee to curb the hunger. We do not have time for lunch.”

“During medical school you do not even have time to grieve, to dream and think about the girlfriend you lost.”

“To have leisure time out of school, time to go to movies, to date, to stay with your parents…to have time for yourself…time to practice sports and eat… to have time to do your things. This is quality of life.”

Table 4 shows other examples of categories and issues for this theme.

5. Coping strategies

**Table 1:** Categories and issues for the theme, “What a medical student’s life is like” from focus groups

**CATEGORY**	**ISSUES**	**EXAMPLES**
Power and status of the profession	Status	“There is a power in the profession, which has an effect on patients’ lives. This power is theoretical and practical; thus, some people feel as if they were demigods.”
	Omnipotence	“We deal with life, as if we were God!”
Experience of disease, pain, suffering, death	Humanitarianism	“I think we should have a more humanistic training.”
	Understanding of the social reality	“You cannot solve the social problems that cause the disease.”
Fear/insecurity	Professional perfomance	“Can I manage it? I don’t know anything.”
Changes in lifestyle	Adaptation difficulties	“I entered the program, moved to town. I live alone. I did not feel myself the same.”
Competition	In training, residency, and professional life	“The competition is big for monitoring, clerkship, and residency.”
Immaturity	Age	“I started the program at the age of 17.”
	Getting mature	“So, I think that it makes you mature quicker.”
	Responsiblity	“I have responsibilities as a student that I never had.”
Curriculum	Contents	“You have to study, there’s much content…”
	Methods	“I think one difference is that in PBL they have time and do not know what to study. In the traditional method we know what to study, but do not have time.”
	Assessments	“Nobody can be happy with assessments every day.”
Models	Teachers	“I want to be like Patch Adams.”
Satisfaction	Personal satisfaction times sacrifices	“It is like having a baby, you know? The baby steals your personal life, your time, your girlfriend, your hours of sleep and occupies space in your house. However, you are completely crazy for him or her. This is the same feeling in medical school, it is great.”
		“You look like hell’ (my friend said)…and I answered, ‘Thanks! I did not sleep last night and was in college the whole day.’ Well, she is getting married and I don’t even have time to get a boyfriend.”

**Table 2:** Categories and issues for the theme, “Concepts of quality of life for medical students” from focus groups

**CATEGORY**	**ISSUES**	**EXAMPLES**
Satisfaction/happiness	Achieving aims	“Quality of life is being happy.”
	Positive vision of life	
Freedom	Choices	“Being able to choose what I want to do.”
Balance/harmony	Work and leisure in harmony	“Having quality of life is having balance and harmony.”
Health	Sleep	“Quality of life is being able to sleep well, eat well, have good moments of leisure.”
	Eating habits	
	Physical activity	
Free time	Leisure, relationships, study, physical activity	“Quality of life is being able to manage time well.”
Interrelation of several life aspects	Social, affective, work, and health	“Quality of life is being able to accomplish my project of life.”

**Table 3:** Categories and issues for the theme, “Factors that increase quality of life in the medical program” from focus groups

**CATEGORY**	**ISSUES**	**EXAMPLES**
Feeling useful	Acknowledgment	“Contact with patients makes you feel useful and contextualizes the study.”
Satisfaction	Having pleasure in activities	“Sometimes you have to work the whole day long without eating. But you learn a lot of things, you live, you get enthusiastic. Then it is all happiness. You extract happiness from the moment when you are doing what you like to do, what you have always wanted to do. In medical school any effort is worthwhile. At that moment I consider my quality of life to be good.”
Significant relationships	Friends, boyfriend/girlfriend, family	“Good relationships within medical school increases quality of life.”
		“Having breakfast with my mother increases my quality of life.”
Time management	Organization	“You should think: I am not only a medical student; I am a citizen with a girlfriend, family and friends. If you recognize that, you will organize better your time, you will try to do things you like at the right moment and try not to demand so much from yourself.”
Significant experiences	Personal development	“Medical school promotes personal growth.”
Teachers	Good examples	“Good teachers increase quality of life.”
Methodology	PBL	“When you do not know the PBL method your quality of life decreases. When you get adapted to it, quality of life increases.”
	Extracurricular activities	“Participating in research projects and in transformation of society increases my quality of life.”

**Table 4:** Categories and issues for the theme, “Factors that decrease quality of life in the medical program” from focus groups

**CATEGORY**	**ISSUES**	**EXAMPLES**
Human resources	Teachers	“Some people hate school because teachers are terrible, they are not teaching and do not know how to teach.”
	Managers	“Managers do not care about our quality of life.”
Moral abuse	Residents and teachers	“There are arrogant teachers.”
Demandings	Personal, familiar, institutional, and social	“The least I can do is to push myself as much as I can do.”
		“Pressure in the medical program is big.”
Eating habits	Inadequate eating habits	“I learned from a resident to put lots of sugar in my coffee to curb the hunger.”
Financial resources	Financial dependence	“You cannot work during the program; I feel bad for being dependent on my parents.”
Lack of care with own health	Students adopt unhealthy life	“You see many medical students getting sick, being down, neglecting one's own body, unable to reflect on these things.”
Sleep	Sleep privacy	“I want, I need to sleep. I cannot do more shifts and study.”
Time	Demands of the program	“I think excessive workload decreases quality of life. You have class every day and then you have to study but you also have many other things in your life.”
		“In the program there’s a lack of quality of life. Lack of time to lose, to think about life, plans, dreams.”

During medical school students may experience varying degrees of difficulty, depending on their coping mechanisms, emerging from moments of crisis. Participants described different strategies to deal with stress and seek out equilibrium during the program. These included practicing sports and engaging in leisure activities such as going to movies, watching TV, listening to music, reading or learning other languages, studying philosophy, looking for meaning in life through religion, and meeting friends to cook and eat. Participants sought support in conversations and discussions with family, friends, and teachers.

“I talk to my friends, I talk to my mother, talk to residents.”

“Conversation therapy, laughter therapy…whatever difficulty you can make into a joke, you try to do it.”

“Food is a social aggregation factor for us.”

Table 5 shows other examples of categories and issues for this theme.

**Table 5:** Categories and issues for the theme, “Coping strategies” from focus groups

**CATEGORY**	**ISSUES**	**EXAMPLE**
Relationships	Talking	“I talk to my friends, I talk to my mother, talk to residents.”
	Mentoring	"The support of my tutor is very important.”
Seeking ways of relaxation / rest	Music and entertainment	“Relaxing, listen to music, lying around doing nothing.”
		“I watch TV programs related to health.”
		“I play the guitar.”
Physical activity	Sports	“We have a very tired mind. It’s good to be physically tired and do sports.”
Knowledge	Search other areas of knowledge	“I read philosophy in my free time.”
		“I travel once a year to get out of the surroundings.”
Social support	Social eating	“Food is a social aggregation factor for us.”
Religiousness	Religion/religious activities as psychological support	“Having religious training increases my quality of life.”

## Discussion

Medical education is characterized by moments of crisis. The first is the initial phase, the adaptation that requires a change in lifestyle and study method. The second crisis occurs in the intermediate phase, when students have contact with reality, extensive content to study, and multiple assessments. The final phase of medical school is characterized by many demands, requirements, and responsibilities, in addition to insecurities that typify the end of the program [11,19]. Such moments may be related to the perception of psychological toxicity in medical school as shown by participants’ reports and the results of previous studies [8,20,21]. Future studies should investigate the causal agents involved to allow for treatment and prevention of this problem.

Use of focus groups comprising medical students allowed us to recognize medical students’ perceptions regarding their quality of life during the medical program and the factors influencing it. This knowledge is of particular importance to the current transformation taking place in medical education in Brazil. Brazil is a developing country that has seen a remarkable social transformation in the past decade, underpinned by macroeconomic stability and rising living standards in the economy, industry, and education [22]. Such development has also reached medical education as Brazil has the second-largest number of medical schools in the world, with 197 institutions and approximately 100,000 medical students [19].

This increase in the number of medical schools has taken place in the last two decades due to social demands for practicing physicians, especially in areas of restricted access such as the Amazon region. However, these emerging schools do not always offer optimal conditions of professional education. This is mostly observed in aspects of psychological support to medical students.

Students reported that their quality of life in medical school is worse than their quality of life perceived in other contexts of their life. However, they evaluated it positively because they perceived sacrifices and difficulties of the program as necessary to attain their goal in life—that is, to become a doctor. Such dissatisfaction is related to the learning environment and the curriculum. However, another likely explanation could be the possible view of students that medical school is a place of “duties” or “work”. In this sense, such dissatisfaction might reflect the current view related to such activities, including study as “duty” or “work”. These duties would be seen as “stolen time”, no longer under the control of the individual, in this case, the student. Considering this fact, the individual feels “robbed” of time that would be his; he cannot serve his own wishes or desires, but must serve others. In the present case the school can be seen as owners of the students’ time [23,24].

Indeed, in the present study participants believed they do not enjoy life as they could. They expressed that they do not experience things appropriate for their age, saying that at the end of graduation they will feel older than their actual age. They also expressed a sense of nostalgia for what was not experienced, without perceiving that being in the program is appropriate for their age. A quantitative study with 800 medical students also found such a lack of time as mentioned by students to be a factor modifying quality of life [11].

We believe medical schools must design curricula with an eye not just toward the medical content but also taking into account students’ lives. A greater effort must be made to decrease competitiveness, and to fight excessive self-demands through discussion groups, mentoring, psychological and pedagogical support. Previous interventional studies have shown these to be effective [20].

It is important to improve the relationship between students and teachers and to value the role of the teacher as an example of how to live with a high quality of life. This is because it is inevitable that the teacher will become a role model for the young person they are training. In addition to imparting knowledge this social and political role of the teacher promotes interaction, guidance, and opinion formation. The life model of the teacher, when professionally and ethically adequate, is transmitted to students with more conviction [25]. We agree with this view and also believe that the teacher can play the role of caregiver, identifying difficulties and supervising students individually.

Medical schools must keep health promotion programs to promote healthy eating habits and physical exercise, as demonstrated in some interventional studies [20,26]. Our reflection on the reports presented here leads us to believe that opportunities must be created for students to participate in social development projects, continuing education, and research. These will provide the student with a feeling of usefulness when contributing to society, producing knowledge, or helping someone. Time management must be dealt with from the beginning of the program because it can strongly minimize stress inherent to many obligations of the program. Indeed, a quantitative study found time management to be an important aspect of medical curricula [11].

Another possibility for improving medical students’ quality of life is to develop students’ resilience with specific content and activities designed throughout the curriculum. Resilience is defined as the capacity to resist adversity in a dynamic process of interaction between social and psychological factors of risk and protection [27-29]. Factors related to resilience are the capacity to recognize problems and the limitations to be faced and communicated; the recording of existing personal resources; the organization and reorganization of one’s action strategies by reviewing one’s current status; the maintenance of creativity, optimism, and a good mood; the ability to be flexible and adapt; and the exercise of responsibility and ethics [27-29]. In the last few years, the concept of resilience has been associated with health promotion, well-being, and quality of life [28].

The concept of resilience can be applied to experiences during the medical program, by seeking to identify activities that stimulate students’ creativity when solving problems or that allow development of responsible autonomy, and relational and decision-making capacity. Among these activities, it is worth mentioning the importance of ludic activities in which the importance of humor in everyday life is recognized, in addition to moments of discussion and reflection that favor self-knowledge and acceptance of one’s capacities and limitations [27].

The results presented here constitute important material for program directors, teachers, and students to reflect on. This is because they point out the need to preserve and improve the health of medical students during this challenging phase of their lives, characterized by difficulties but also the satisfaction of accomplishing one’s life goals.

Our results reflect Brazilian students’ experiences portrayed in their culture and social identity. We cannot assume that students in other cultures hold the same perceptions of quality of life. However, we do believe that most of these feelings may be common among medical students from different countries around the world.

## Conclusions

Experiences in medical school are associated not only with personal and professional development, but also with psychological toxicity and a negative impact on students’ quality of life. Factors influencing medical students’ quality of life include the quality of teachers, curricula, and changes in lifestyle related to eating habits, sleep, and physical activity. Lack of time due to the multifarious obligations of medical school emerged as a major factor. Students’ coping strategies included participation in social networks, physical activities, religious support, mentoring, and development of other areas of knowledge. Students affirmed that their quality of life is influenced by their experiences in medical school, but they also reframe their difficulties represented by their poor quality of life. This is because they understand them as necessary and inherent to the process of becoming doctors.

The results of this study led to the creation of a collaborative network of psychological support to medical students by the Brazilian Association for Medical Education in all regions of Brazil. This collaborative network also fosters research, annual meetings, and implementation of new support services. Our experience leads us to believe that offering better learning environments and psychological support to medical students results in the education of more competent and socially responsible doctors.

## Competing interests

The authors declare that they have no competing interests.

## Authors’ contributions

PT participated in the conception and design of the study, carried out the data acquisition, participated in the analysis and interpretation of data and drafted the manuscript. PLB participated in the data acquisition and analysis and interpretation of the data. HBMSP and SCE critically reviewed the manuscript. MM participated in the conception and design of the study. LBS participated in the conception and design of the study and in the analysis and interpretation of data. All authors read and approved the final manuscript.

## Pre-publication history

The pre-publication history for this paper can be accessed here:

http://www.biomedcentral.com/1472-6920/12/106/prepub
